# One-Step Hydrothermal Synthesis of Nanostructured MgBi_2_O_6_/TiO_2_ Composites for Enhanced Hydrogen Production

**DOI:** 10.3390/nano12081302

**Published:** 2022-04-11

**Authors:** Feng Xu, Chaohao Hu, Di Zhu, Dianhui Wang, Yan Zhong, Chengying Tang, Huaiying Zhou

**Affiliations:** 1 School of Materials Science and Engineering, Guilin University of Electronic Technology, Guilin 541004, China; xufeng118292@163.com (F.X.); heyjude456@163.com (D.Z.); devix@mails.guet.edu.cn (D.W.); yanzhong@guet.edu.cn (Y.Z.); ctang@guet.edu.cn (C.T.); zhy@guet.edu.cn (H.Z.); 2 Guangxi Key Laboratory of Information Materials, Guilin University of Electronic Technology, Guilin 541004, China

**Keywords:** MgBi_2_O_6_/TiO_2_, photocatalyst, heterojunction, hydrothermal method

## Abstract

A highly efficient MgBi2O_6_ (MBO)/TiO_2_ heterostructured photocatalyst for the evolution of H_2_ was successfully prepared using a one-step hydrothermal method. The phase structure, microstructure and optical properties of the MBO/TiO_2_ composites were investigated by various experimental techniques. A series of H_2_ production experiments were performed under visible light. The measured results indicated that the nanostructured MBO/TiO_2_ composite, with a stoichiometric molar ratio of MBO:TiO_2_ = 0.2%, displayed the best H_2_ production rate of 3413 μmol h^−1^ g^−1^. The excellent photocatalytic performance of the obtained composite material was due to the heterojunction formed between MBO and TiO_2_, which reduced the charge transfer resistance and accelerated the separation efficiency of the photogenerated electron–hole pairs. The reaction mechanism was also discussed in detail.

## 1. Introduction

The quick development of science and technology has facilitated peoples’ lives, but has also resulted in an aggravated energy shortage and environmental deterioration. The development of clean fuels is one of the most important and urgent issues in the 21st century. In this regard, semiconductor photocatalysis technology is of great interest, since it can produce clean hydrogen by the direct utilization of solar energy. Since Fujishima and Honda first discovered that, under UV light irradiation, water can be decomposed to produce H_2_, using a TiO_2_ electrode, numerous studies on TiO_2_ have been conducted in detail to explain the reaction mechanism and to improve the photocatalytic efficiency [1,2]. TiO_2_ has been considered the most widely investigated photocatalyst for H_2_ production because it possesses some significant advantages, such as superior stability, non-toxicity, and low cost [3,4]. However, the more widespread application of TiO_2_ in photocatalysis is greatly restricted, due to the low quantum yield and low efficiency of sunlight utilization, mainly resulting from its relatively wide bandgap of about 3.2 eV [5,6,7]. Thus, it is imminently necessary to probe novel compounds with outstanding photocatalytic properties under visible light irradiation [8,9].

In recent years, bismuth-based semiconductors, as one kind of new photocatalytic material, have been widely studied [10,11]. These compounds usually contain trivalent or pentavalent states of bismuth. It is worth noting that bismuth-based semiconductor photocatalysts possess a unique electronic structure. A continuously uplifted valence band, which reduces the band gap, is formed, due to the hybridization of O-2p and Bi-6s orbitals [12]. Bi^5+^ has a different electronic structure from the trivalent bismuthate, with an empty 6s orbital, but still has a d10 blocking shell [13]. The light absorption range of these materials is expanded, due to this unique feature, which leads to improved photocatalytic performance. Among the bismuth-based compounds, MgBi_2_O_6_ (MBO), with its trirutile-type structure, can be considered a visible light-responsive photocatalyst, which possesses a relatively small band gap of 1.8 eV [14]. However, compared with other well-studied photocatalysts, the photocatalytic efficiency of MBO is relatively low, which may be due to its low redox ability, mainly resulting from the narrow band gap.

Currently, one of the most valid and executable pathways to obtain a new material with significantly enhanced photocatalytic performance is to construct a semiconductor heterojunction, through combining semiconductors with different band gaps [15,16,17]. The synthesized heterostructured composites generally possess obviously enhanced photocatalytic activity, since the photoinduced electrons and holes are effectively separated and transferred to the heterojunction under the driving force of the internal electric field, and plenty of holes and electrons participating in redox reactions would be generated [18,19]. Many heterojunctions have been applied to promote photocatalytic reactions, and TiO_2_-based heterojunction photocatalysts, in particular, have been widely studied. For instance, Peng and co-authors reported that AgIn_5_S_8_/TiO_2_ composites exhibited significantly enhanced photocatalytic performance in the production of H_2_ because of the heterojunction structure built between AgIn_5_S_8_ and TiO_2_ [20]. Currently, MBO/TiO_2_ composites have not been reported, and the improvement in photocatalytic H_2_ production of TiO_2_, induced by the addition of MBO, needs to be investigated in detail.

In the present paper, we successfully synthesized the MBO/TiO_2_ composite photocatalysts using a one-step hydrothermal method. Compared with pure MBO and TiO_2_, the photocatalytic performance of the MBO/TiO_2_ composites clearly improved and the H_2_ production rate under visible light significantly improved. The corresponding photocatalytic reaction mechanism was discussed further. 

## 2. Materials and Methods

### 2.1. Synthesis of MBO Nanospheres and MBO/TiO_2_ Photocatalysts

All synthetic chemicals were analytically pure and required no additional purification. Pure MBO was synthesized using a hydrothermal method. NaBiO_3_•5H_2_O (x = 0.025, 0.1, 0.2, 0.3, and 1.5 mmol) was dissolved in 30 mL of water with about 10 min of strong stirring to obtain solution A. MgCl_2_•2H_2_O (4x mmol) was dispersed in 30 mL of pure water and stirred for about 10 min to obtain solution B. Then, the orange mixed solution was obtained by mixing solution A with solution B and vigorously stirring for 30 min. Under the stirring state, 0.7987 g of TiO_2_ (Degussa P25, Frankfurt, Germany) was gradually added to the mixed solution. The pH value of the mixture was kept at 8.5 by adding 4 mol/L of NaOH solution. The mixture was kept at 130 °C for 6 h in a 100 mL stainless steel autoclave. After this, the mixture in the autoclave was filtered to obtain the crude product. The final product was obtained via washing with pure water, as well as with absolute ethanol, several times, and then dried at 60 °C for 12 h. In this article, the molar ratios of MBO to TiO_2_ were 0.01%, 0.05%, 0.2%, 0.6% and 3%, which were represented as 0.01MBO/TiO_2_, 0.05MBO/TiO_2_, 0.2MBO/TiO_2_, 0.6MBO/TiO_2_ and 3MBO/TiO_2_, respectively.

### 2.2. Characterization of Samples

The X-ray diffractometer (XRD) (D8 Advance, Bruker, Billerica, MA, USA) was used to obtain the physical phase and used in the purity analysis of samples. The morphologies and microstructures of MBO, TiO_2_ and MBO/TiO_2_ were characterized using a field emission scanning electron microscope (FESEM) (FEI Quanta 450 FEG, Hillsboro, OR, USA) and a transmission electron microscope (TEM) (FEI Tecnai G20, ThermoFisher, Waltham, MA, USA). The X-ray photoelectron spectroscopy (XPS) (Thermo ESCALAB 250Xi, ThermoFisher, Waltham, MA, USA) was employed to investigate the elemental compositions and oxidation states of the MBO/TiO_2_ composites. The ultraviolet–visible (UV–Vis) spectrometer (Puxi TU-1901, PERSEE, Beijing, China) was used to measure the UV–Vis diffuse reflectance spectroscopy (UV–Vis DRS) of the as-prepared samples, with barium sulfate as the reference. The photocatalytic analysis system (Labsolar-IIIAG, Perfect-light, Beijing, China) was used to examine the production of H_2_.

### 2.3. Photocatalytic Hydrogen Production Test

A 300 W Xe irradiation lamp was placed on the top of the photocatalytic hydrogen production reactor, which was connected to the Labsolar-III (AG) system. In a general test, 0.1 g of the heterostructured composite catalysts was added to the solution, with methanol serving as the sacrificial agent. The irradiation started to work after the air in the system was thoroughly eliminated. An online gas chromatograph (GC7900, Techcomp, Beijing, China) was used to periodically analyze the hydrogen generated from the photocatalytic reaction. The stability of the photocatalyst was checked throughout the experiment cycle. Following the same steps, it was tested 5 times under visible light. Before the reaction started, the system was evacuated and purified with nitrogen to make sure that there was no H_2_ or O_2_.

### 2.4. Photoelectrochemical Studies

The photochemical tests were carried out using an electrochemical system (CHI-660B, Tianjin, China). A three-electrode system, with a Na_2_SO_4_ electrolyte solution (0.1 M), was used in the measurements, in which the synthesized photocatalyst was firstly uniformly plated on the fluorine-doped SnO_2_ conductive (FTO) glass sheet with ethanol ultrasonication, and then the binder was added dropwise, followed by drying the plated FTO glass sheet in an oven. After completion, it was removed and used as the working electrode. The saturated calomel electrode was used as the reference electrode and the platinum wire served as the counter electrode.

## 3. Results and Discussion

### 3.1. Phase Structure and Morphological Analysis

**Figure 1:**
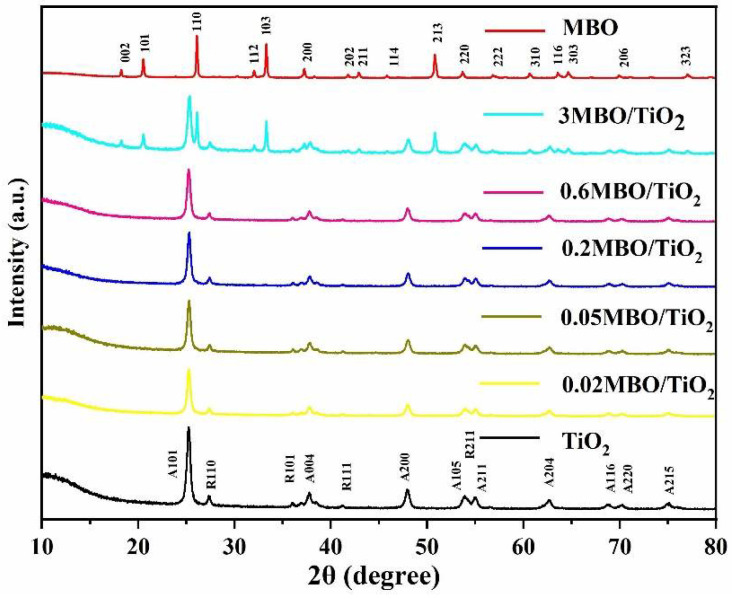
XRD patterns of pure MBO, TiO_2_ and the as-prepared MBO/TiO_2_ composites with different MBO contents.

The crystallinity and composition of MBO, TiO_2_ and the MBO/TiO_2_ composites, synthesized at 130 °C with a range of molar ratios of MBO to TiO_2_, were measured using XRD, and are shown in Figure 1. The XRD peaks at the 2θ values of 18.2°, 20.5°, 26.1°, 32°, 33.3°,37.2°, 50.8°, 53.7° and 64.6° correspond to the (002), (101), (110), (112), (103), (200), (213), (220) and (303) crystal surfaces of pure MBO, respectively. This matches well with the standard card (JCPDS No.86-2492) of MBO. The characteristic peaks of the rutile and anatase phases of TiO_2_ can be found in Figure 1, and are well indexed to the standard XRD patterns (JCPDS No.21-1276 and No.21-12). No other crystal phases were found from the XRD patterns of MBO/TiO_2_, indicating that the obtained composite material has high purity. It is obvious that, with the increase in the MBO component, the intensities of the XRD peaks of MBO become stronger and the characteristic diffraction peaks of TiO_2_ would gradually become weaker. In addition, when the amount of added MBO is less than 0.6%, there are no diffraction peaks of MBO observed. The XRD patterns of 0.2 MBO/TiO_2_ indicate that the structures of TiO_2_ and MBO remain stable during the preparation of MBO/TiO_2_. The sharp and intense diffraction peaks indicate that the product crystallizes very well. The XRD results clearly demonstrate the formation of MBO/TiO_2_ composites with good crystallinity via the hydrothermal method.

**Figure 2:**
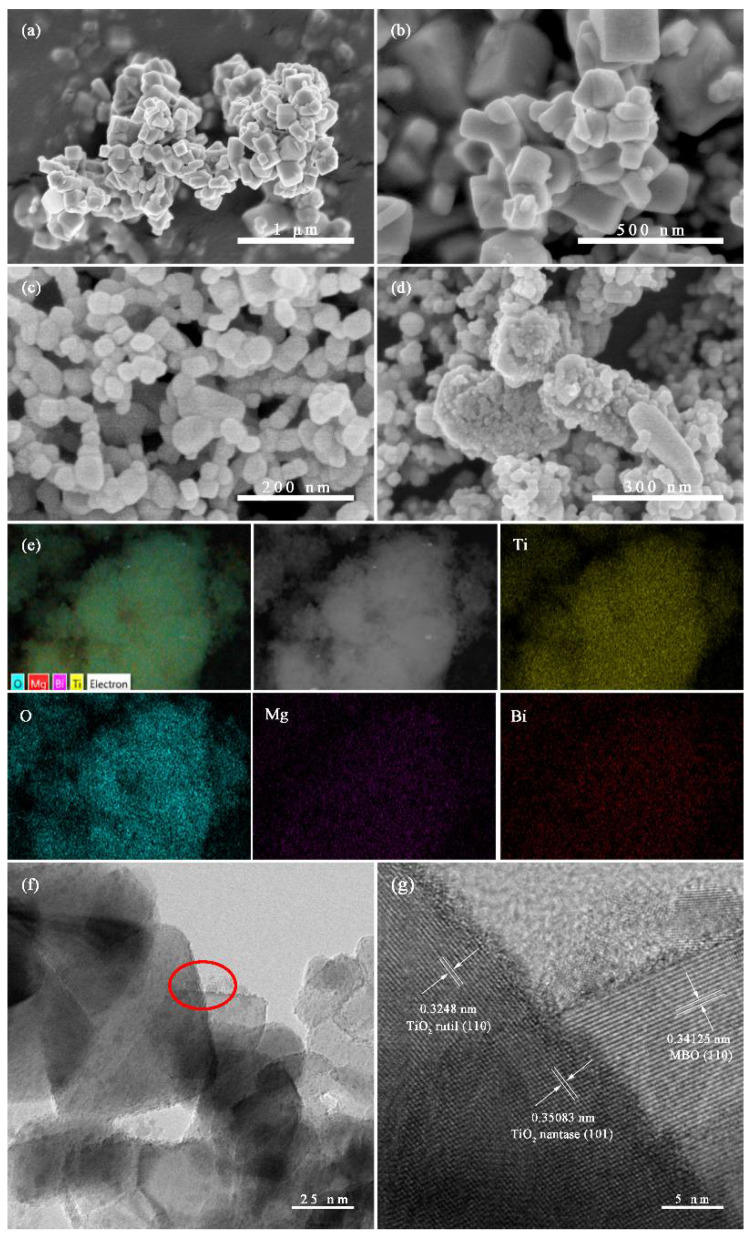
SEM images of (**a**,**b**) MBO, (**c**) TiO_2_, (**d**) 0.2 MBO/TiO_2_, (**e**) EDS elemental mapping images, (**f**) TEM and (**g**) HRTEM images of 0.2 MBO/TiO_2_.

The measured morphologies and microstructures of pure MBO, TiO_2_ and the prepared 0.2 MBO/TiO_2_ composites from SEM are placed in Figure 2. It can be observed from Figure 2a,b that pure MBO consists of irregular hexahedron morphologies, with a length and width of about 80~250 nm. The morphology of pure TiO_2_, as shown in Figure 2c, is composed of irregular cubic particles, with a size of about 20–50 nm. For the 0.2 MBO/TiO_2_ composite shown in Figure 2d, one can observe that the morphology of MBO is transformed from the original hexahedral particles into irregular cubic particles, and a large number of small TiO_2_ nanoparticles are homogeneously distributed around the MBO particles. Figure 2e shows the EDS mapping images of the 0.2 MBO/TiO_2_ composite nanomaterials, visualizing the distribution of Ti, O, Mg and Bi. The detailed microstructure of the 0.2 MBO/TiO_2_ nanocomposite can be further observed by TEM and high-resolution TEM (HRTEM). Figure 2f shows that MBO is in close contact with TiO_2_ particles, with a size of about 30–120 nm. It can be found, from the measured lattice fringes of the composite shown in Figure 2g, that the tested lattice parameters are about 0.3516, 0.2187, and 0.3412 nm, and correspond to the (101) plane of anatase TiO_2_, the (111) plane of rutile TiO_2_ [21], and the (110) plane of MBO, respectively. The interface between TiO_2_ and MBO nanoparticles is clear, and can provide a reaction center for the reaction [22]. For the present study, only two components of MBO and TiO_2_ formed in the heterojunction, without other impurities.

**Figure 3:**
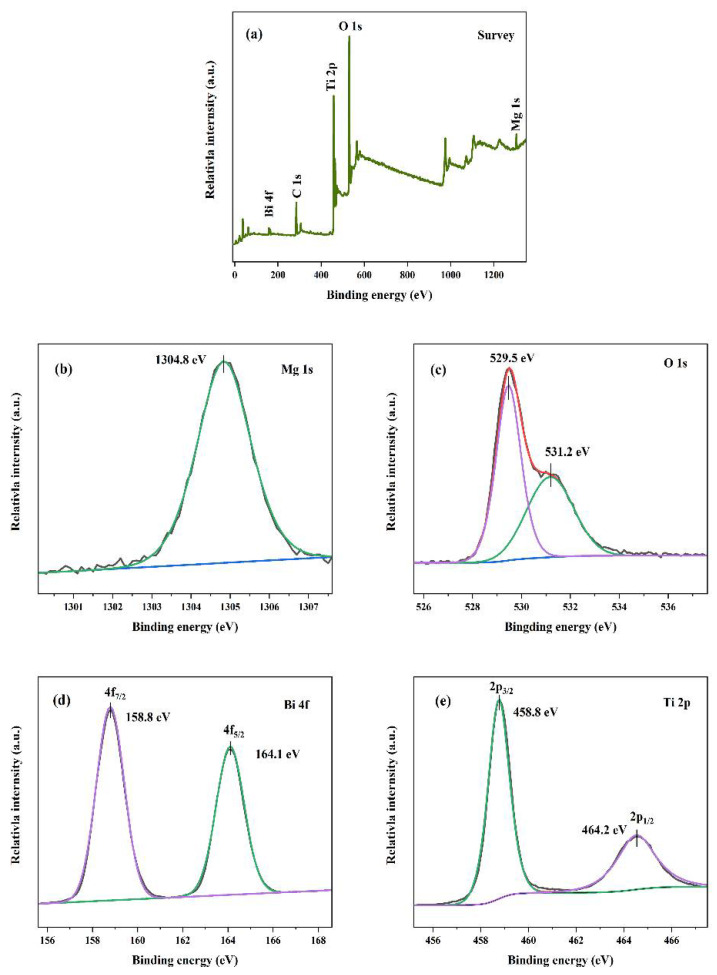
XPS spectra of 0.2 MBO/TiO_2_: (**a**) full scan survey of all the elements; (**b**) Mg1s; (**c**) O1s; (**d**) Bi4f; (**e**) Ti2p.

To evaluate the elemental status and chemical composition of the prepared samples, the 0.2 MBO/TiO_2_ catalyst was further measured using XPS. The full-scan spectrum of 0.2 MBO/TiO_2_, as presented in Figure 3a, confirms the existence of Ti, O, Mg, Bi and C elements. Generally, the adventitious hydrocarbons generated from the XPS instrument are considered to be the cause of the occurrence of the C1s peak at about 284.7 eV in the XPS full spectrum. Figure 3b shows that the peak at around 1303.8 eV can be assigned to the Mg1s peak of Mg^2+^ [23]. The peaks at 529.5 eV and 531.2 eV in Figure 3c correspond to the lattice oxygen of the Ti-O/Bi-O bond in O1s, and the chemically adsorbed oxygen [21], respectively. The peaks at around 164.1 eV and 158.5 eV, presented in Figure 3d, correspond to Bi 4f_5/2_ and Bi 4f_7/2_, confirming the presence of the Bi^5+^ state [14,24]. In Figure 3e, there are two peaks at around 458.8 eV and 464.2 eV, which can be indexed to Ti 2p_3/2_ and Ti 2p_1/2_, respectively [18], and confirm the presence of Ti^4+^ cations in the MBO/TiO_2_ composites [25]. As a result, the measured XPS results have proved the successful synthesis of the MBO/TiO_2_ heterostructure.

**Figure 4:**
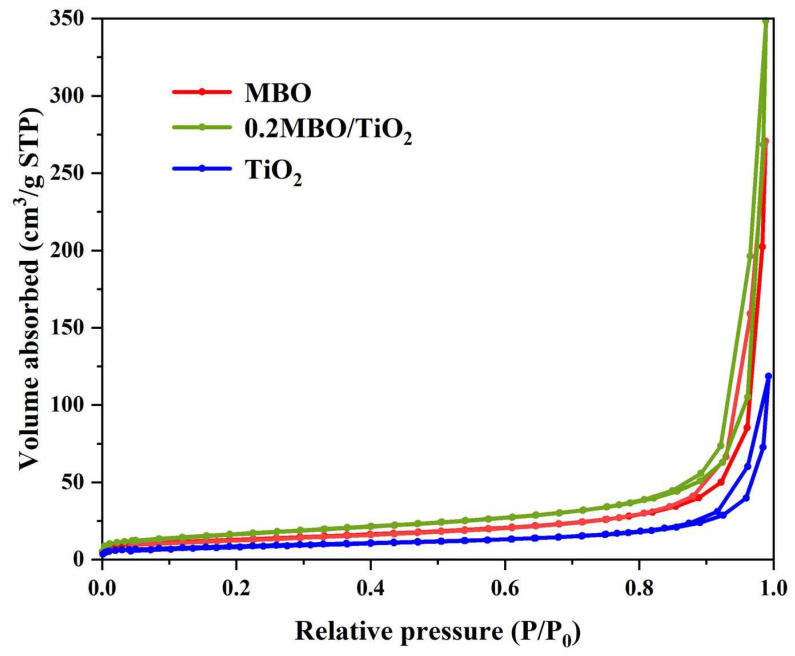
N_2_ isothermal adsorption and desorption test curves of MBO, 0.2 MBO/TiO_2_ and TiO_2_.

The specific surface area is a factor that affects the catalytic activity of photocatalytic materials, and the specific surface area is generally measured using the N_2_ isothermal adsorption and desorption curves. As shown in Figure 4, the N_2_ isothermal adsorption and desorption curves of MBO, 0.2 MBO/TiO_2_ and TiO_2_ were 45.94 cm^2^ g^−1^, 59.69 cm^2^ g^−1^ and 30.87 cm^2^ g^−1^, respectively. The measured results show that the prepared 0.2 MBO/TiO_2_ composites with larger specific surface areas are expected to possess higher catalytic activity, in comparison with MBO and TiO_2_.

### 3.2. Light Absorption Spectra of MBO/TiO_2_ Catalysts

**Figure 5:**
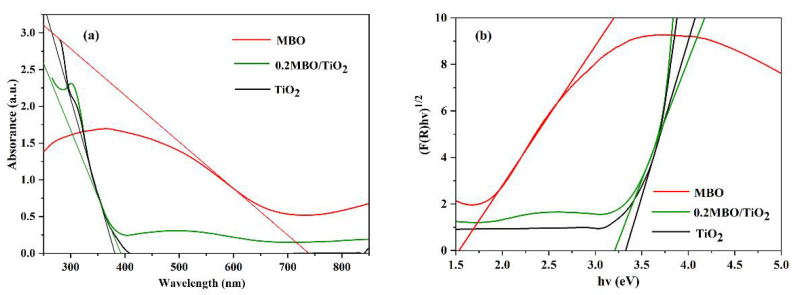
(**a**) UV–Vis DRS and (**b**) the plots of (*F*(*R*)*hv*)^1/2^ versus *hv* of MBO, TiO_2_ and 0.2 MBO/TiO_2_.

The absorption spectra of pure MBO, TiO_2_ and the 0.2 MBO/TiO_2_ composite photocatalysts were measured using a UV–Vis spectrophotometer, and are presented in Figure 5. We can observe that the absorption edges of pure TiO_2_ and MBO are at about 392 nm and 756 nm. A slight redshift of the absorption spectrum of the 0.2 MBO/TiO_2_ composite photocatalysts can be found in comparison with pure TiO_2_, and its light absorption band edge appears in the visible area greater than 400 nm, which is due to the sensitization of the narrow bandgap structure of MBO [26,27]. The following equation can be used to calculate the energy band gap (*E*_g_) of MBO, TiO_2_ and MBO/TiO_2_:

(*F*(*R*)*hv*)^1/*n*^ = *A*(*hv* − *E*_g_) (1)

where *F*(*R*), *v*, *h* and *A* represent the diffuse absorption coefficient, optical frequency, Planck’s constant and proportionality constant, respectively. The value of *n* is related to the transition type of the semiconductor (here, *n* = 2 corresponds to the indirect semiconductor in this article). The *E*_g_ values of 0.2 MBO/TiO_2_, MBO and TiO_2_ are 3.1 eV, 1.65 eV and 3.2 eV, respectively. The calculated values for MBO and TiO_2_ are similar to the previous reports [14,28]. The *E*_g_ of 0.2 MBO/TiO_2_ is slightly smaller than that of TiO_2_, which indicates that the MBO/TiO_2_ composites would have a wider light response range, due to the addition of MBO with a small *E*_g_ value.

**Figure 6:**
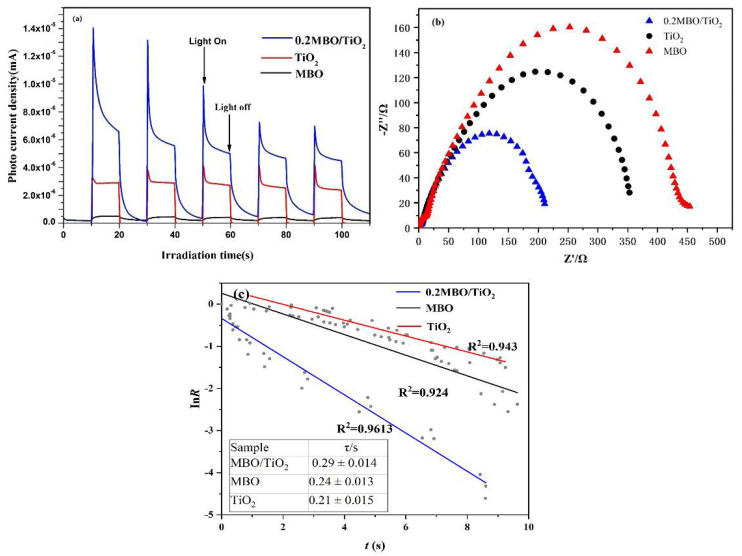
(**a**) Transient photocurrent curves, (**b**) EIS plots, and (**c**) normalized plots of current–time dependence of the MBO, TiO_2_ and 0.2 MBO/TiO_2_ catalysts, together with the corresponding transient time constants, as shown in the inserted table.

The measurements of the transient photocurrent response of pure TiO_2_, MBO and 0.2 MBO/TiO_2_ composite materials, and the corresponding electrochemical impedance spectroscopy (EIS), are helpful for fully understanding the transmission and separation of photogenerated carriers in photocatalysts [22]. Figure 6a clearly shows that the 0.2 MBO/TiO_2_ heterostructured composite has a much higher transient photocurrent density compared to pure MBO and TiO_2_. This proves that the formation of the MBO/TiO_2_ heterojunction accelerates the separation of photogenerated charges and significantly enhances the photocatalytic performance of the composite catalyst. The EIS measurement is then performed to confirm the charge transfer resistance, and the arc radius of the EIS plots can reveal the response rate. The separation of the electron–hole pair and the photocatalytic reaction is faster when the radius of the impedance spectrum arc is smaller. The impedance plot presented in Figure 6b shows that the 0.2 MBO/TiO_2_ heterostructured composite has a semicircle with a smaller arc, in comparison with pure MBO and TiO_2_, suggesting that the addition of MBO to TiO_2_ is beneficial for separating the electron–hole pairs, owing to the reduction in charge transfer resistance; this facilitates the obvious enhancement in the photocatalytic performance of the 0.2 MBO/TiO_2_ composite. The normalized plot of the transient photocurrent presented in Figure 6c shows that the transient time constant of the 0.2 MBO/TiO_2_ composite is higher than that of MBO and TiO_2_, indicating a slower recombination rate [29].

### 3.3. Hydrogen Production Performance

**Figure 7:**
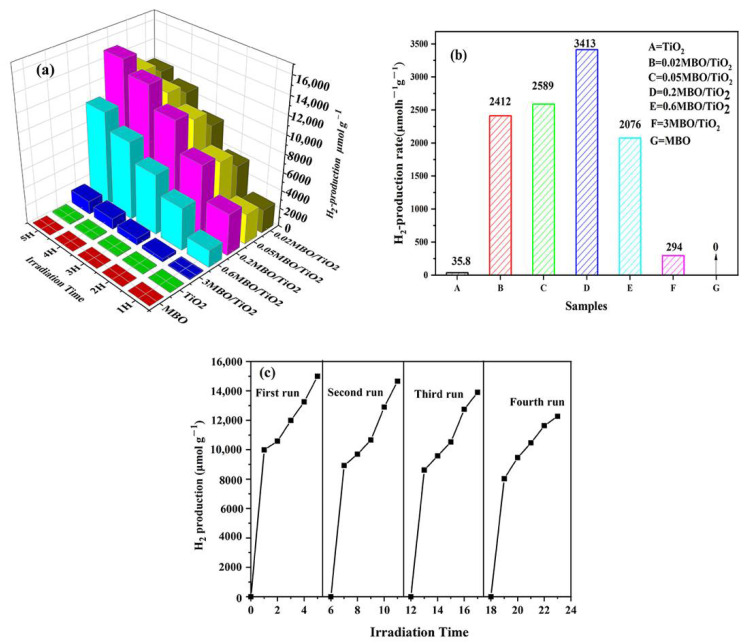
(**a**) Photocatalytic hydrogen generation, (**b**) the hydrogen generation rate of different samples under visible light irradiation, and (**c**) recycling tests for photocatalytic hydrogen generation of 0.2 MBO/TiO_2_.

The measurements of hydrogen production were carried out by adding 0.1 g of catalyst powder to an 80 mL solution, consisting of 20 mL of methanol and 60 mL of deionized water. Figure 7a shows the amount of photocatalytic hydrogen production for the MBO/TiO_2_ composite catalysts under 5 h of visible light irradiation. We can observe that the hydrogen production gradually increases and then decreases with the increase in MBO content. The best loading amount of MBO is about 0.2% and the maximal H_2_ production is 15,007 μmol·g^−1^ in 5 h, which is nearly 80 times higher than the hydrogen production of pure TiO_2_. Figure 7a also shows that pure MBO is not capable of photocatalytic H_2_ evolution. The decrease in H_2_ production in the MBO/TiO_2_ composites, with a higher MBO content, is probably due to the weakness of catalytic activity on the surface of TiO_2_, since the incident light is blocked and the production of electrons by TiO_2_ is suppressed because of the high loading amount of MBO [30,31,32,33]. The results of the hydrogen production rate of MBO/TiO_2_ are shown in Figure 7b. The hydrogen production rate of 0.2 MBO/TiO_2_ is 3413 μmolh^−1^g^−1^, which clearly exceeds that of the samples with other components and single phases. The cycle measurements for MBO/TiO_2_ were carried out to confirm the stability of the photocatalyst. The results presented in Figure 7c show that, after the fourth cycle, the H_2_ production is 12,275 μmol·g^−1^, and is about 82% of the first cycle. This indicates the good stability of the 0.2 MBO/TiO_2_ photocatalyst. The possible reason for the slight decrease in hydrogen production is that the HCOOH formed by the decomposition of CH_3_OH slightly stimulates the activity of the photocatalyst and affects the sensitivity of the photocatalyst to light. Moreover, it can be found from Table 1 that the hydrogen production activity of MBO/TiO_2_ is comparable to that of other TiO_2_-based heterojunctions, as reported in previous literature.

### 3.4. Possible Mechanism of Hydrogen Production

**Figure 8:**
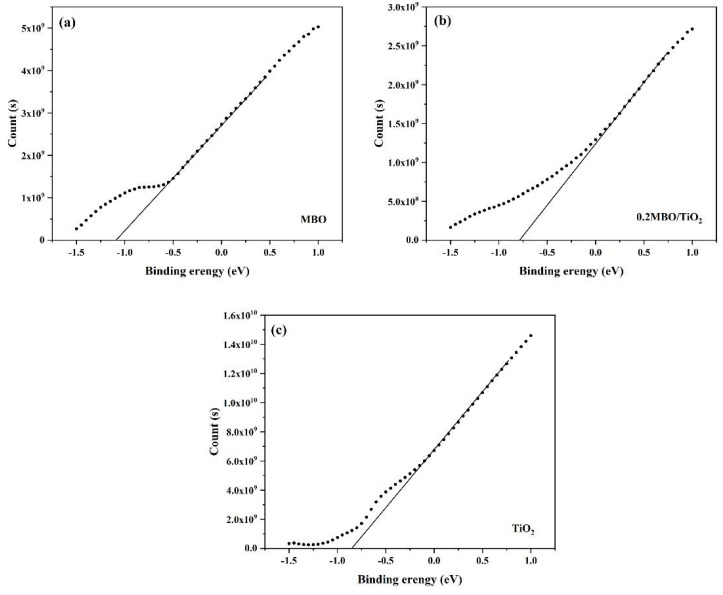
Mott–Schottky plots for (**a**) MBO, (**b**) 0.2 MBO/TiO_2_ and (**c**) TiO_2_.

Figure 8 shows the Mott–Schottky curves of MBO, 0.2 MBO/TiO_2_ and TiO_2_ at 2000 Hz. The corresponding flat-band potentials are −1.1 eV, −0.78 eV and −0.85 eV, respectively. All three catalysts have positive slopes on the Mott–Schottky plots, indicating that they all belong to the *n*-type semiconductor. In addition, the flat-band potential of 0.2 MBO/TiO_2_ shifts to a less negative value, in comparison with those of MBO and TiO_2_ in the single phase. This is also evidence of the formation of an *n*-*n* type heterojunction between MBO and TiO_2_.

**Figure 9:**
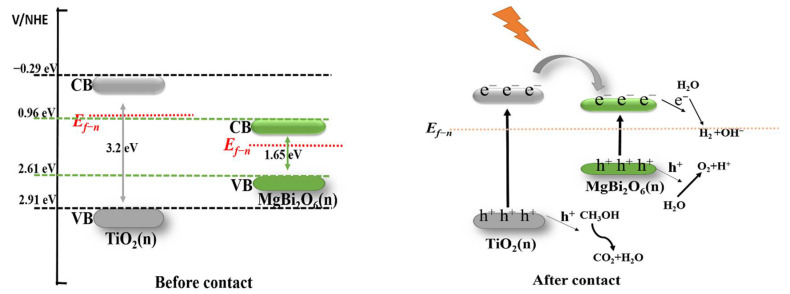
Schematic energy band diagrams of the MBO/TiO_2_ *n*-*n* heterojunction and the possible photocatalytic mechanism of hydrogen production.

The potential mechanism of photocatalytic hydrogen production for MBO/TiO_2_ composite catalysts was investigated further, and can be clearly described using a schematic energy band diagram, as shown in Figure 9. First, the edge potential of the conduction band (ECB) and that of the valence band (EVB) for MBO and TiO_2_ can be calculated using the following equations [25]:

*E*_CB_ = 0.5*E*_g_ + *χ* − *E*_e_

*E*_VB_ = *E*_g_ + *E*_CB_

Among them, *χ* is the electronegativity of the semiconductor (6.28 eV for MBO and 5.81 eV for TiO_2_). *E*_e_ (approximatively 4.5 eV) represents the energy of the free electrons in the standard hydrogen electrode [32]. After calculation, the values of *E*_VB_ and *E*_CB_ are 0.96 and 2.61 eV for MBO, and −0.27 and 2.9 eV for TiO_2_, respectively.

Based on the above results and considerations, we proposed a possible hydrogen production reaction mechanism for the MBO/TiO_2_ composite photocatalytic system. Since the bottom of the conduction band of TiO_2_ is higher than MBO, the corresponding top of the valence band is lower than MBO, as shown in Figure 9. On the one hand, when the material is exposed to light, the electrons in the valence band are excited to transfer to the conduction band, and, at the same time, the holes in the valence band are generated [38]. Due to the potential difference between them, the photogenerated electrons are transferred from the conduction band of TiO_2_ to the conduction band of MBO, and they react with water to generate H_2_ and OH^-^ on the surface of the catalyst. On the other hand, the holes in the valence band can directly oxidize water to release O_2_ and H^+^. At the same time, the sacrificial agent methanol will react with OH^-^ and H^+^, under the action of h^+^, to produce CO_2_ and H_2_O, and other non-polluting substances. However, CO_2_ is easily soluble in water and is difficult to detect. The heterojunction is an important basis for photocatalysis. We believe that the MBO/TiO_2_ composite photocatalytic system can exhibit excellent photocatalytic activity, due to the rapid separation of photogenerated electron–hole pairs, which is accelerated by the MBO/TiO_2_ heterojunction.

## 4. Conclusions

A new MBO/TiO_2_ heterojunction composite material, with the highest efficiency for photocatalytic hydrogen evolution, was prepared successfully through a one-step hydrothermal method. The photocatalytic performance of the MBO/TiO_2_ composite is clearly superior to that of the single-component material, under visible light. Particularly, it exhibits excellent stability and the highest efficiency of hydrogen production when the molar ratio of MBO to TiO_2_ is 0.2%. From the test results and the above-mentioned discussion of the photocatalytic mechanism, it is evident that the separation and transfer of photoinduced electrons and holes are promoted by the formation of the MBO/TiO_2_ *n*-*n* heterojunction, while the electron–hole pairs hardly recombine, which is a key factor in improving the photocatalytic activity.

## Figures and Tables

**Table 1:** A comparison of different TiO_2_ composite photocatalysts for hydrogen production.

Photocatalyst	Light Source	Reactant Solution and Sacrificial Reagents	H_2_ Evolution Rate	Ref.
(Sr_0_._6_Bi_0_._305_)_2_Bi_2_O_7_/TiO_2_	PLS-SXE 300	Methanol aqueous solution	3.18 mmol h^−1^ g^−1^	[28]
AgIn_5_S_8_/TiO_2_	300 W Xe lamp	Na_2_SO_3_ and Na_2_S aqueous solution	371.1 μmol h^−1^	[20]
CdS/TiO_2_	350 W Xenon lamp	Na_2_S aqueous solution	2885 μmol h^−1^ g^−1^	[34]
SnO_2_/TiO_2_	Ultraviolet light (UV)	Na_2_S and Na_2_SO_3_ aqueous solution	150 μmol h^−1^ g^−1^	[35]
TiO_2_/g-C_3_N_4_	300 W Xenon arc lamp	Triethanolamine aqueous solution	39.18 mmol h^−1^ g^−1^	[36]
TiO_2_/ZnIn_2_S_4_	300 W Xenon lamp	Lactic acid aqueous solution	4958 μmol h^−1^ g^−1^	[37]
MBO/TiO_2_	PLS-SXE 300	Methanol aqueous solution	3413 μmol h^−1^ g^−1^	This work

## Data Availability

Not applicable.

## References

[1] Fujishima A., Honda K. (1972). Electrochemical photolysis of water at a semiconductor electrode. Nature.

[2] Mills A., Davies R.H., Worsley D. (1993). Water-purification by semiconductor photocatalysis. Chem. Soc. Rev..

[3] Fox M.A., Dulay M.T. (1993). Heterogeneous photocatalysis. Chem. Rev..

[4] Hoffmann M.R., Martin S.T., Choi W.Y., Bahnemann D.W. (1995). Environmental applications of semiconductor photocatalysis. Chem. Rev..

[5] Zou Z.G., Ye J.H., Sayama K., Arakawa H. (2001). Direct splitting of water under visible light irradiation with an oxide semiconductor photocatalyst. Nature.

[6] Kudo A., Miseki Y. (2009). Heterogeneous photocatalyst materials for water splitting. Chem. Soc. Rev..

[7] Chen C.C., Ma W.H., Zhao J.C. (2010). Semiconductor-mediated photodegradation of pollutants under visible-light irradiation. Chem. Soc. Rev..

[8] Linic S., Christopher P., Ingram D.B. (2011). Plasmonic-metal nanostructures for efficient conversion of solar to chemical energy. Nat. Mater..

[9] Meng X.C., Li Z.Z., Chen J., Xie H.W., Zhang Z.S. (2018). Enhanced visible light-induced photocatalytic activity of surface-modified BiOBr with Pd nanoparticles. Appl. Surf. Sci..

[10] Cheng L.J., Kang Y. (2013). Synthesis and characterization of Bi_2_O_3_/NaBiO_3_ composite visible light-driven photocatalyst. Mater. Lett..

[11] Shen J., Wang R., Liu Q.Q., Yang X.F., Tang H., Yang J. (2019). Accelerating photocatalytic hydrogen evolution and pollutant degradation by coupling organic co-catalysts with TiO_2_. Chin. J. Catal..

[12] Kako T., Zou Z., Katagiri M., Ye J. (2007). Decomposition of organic compounds over NaBiO_3_ under visible light irradiation. Chem. Mater..

[13] Takei T., Haramoto R., Dong Q., Kumada N., Yonesaki Y., Kinomura N., Mano T., Nishimoto S., Kameshima Y., Miyake M. (2011). Photocatalytic activities of various pentavalent bismuthates under visible light irradiation. J. Solid State Chem..

[14] Zhong L.S., Hu C.H., Zhuang J., Zhong Y., Wang D.H., Zhou H.Y. (2018). AgBr/MgBi_2_O_6_ heterostructured composites with highly efficient visible-light-driven photocatalytic activity. J. Phys. Chem. Solids.

[15] Zhu D., Wang X.L., An H.T., Zhong Y., Wang D.H., Tang C.Y., Hu C.H. (2020). Facile one-step hydrothermal fabrication of (Sr_0.6_Bi_0.305_)_2_Bi_2_O_7_/SnO_2_ heterojunction with excellent photocatalytic activity. Nanomaterials.

[16] Meng S., Zhang J., Chen S., Zhang S., Huang W. (2019). Perspective on construction of heterojunction photocatalysts and the complete utilization of photogenerated charge carriers. Appl. Surf. Sci..

[17] Hu C.H., Zhuang J., Zhong L.S., Zhong Y., Wang D.H., Zhou H.Y. (2017). Significantly enhanced photocatalytic activity of visible light responsive AgBr/Bi_2_Sn_2_O_7_ heterostructured composites. Appl. Surf. Sci..

[18] Nanu M., Schoonman J., Goossens A. (2005). Solar-energy conversion in TiO_2_/CuInS_2_ nanocomposites. Adv. Funct. Mater..

[19] Ju P., Wang Y., Sun Y., Zhang D. (2016). Controllable one-pot synthesis of a nest-like Bi_2_WO_6_/BiVO_4_ composite with enhanced photocatalytic antifouling performance under visible light irradiation. Dalton Trans..

[20] Li K., Xu J.L., Zhang X.H., Peng T.Y., Li X.G. (2013). Low-temperature preparation of AgIn_5_S_8_/TiO_2_ heterojunction nanocomposite with efficient visible-light-driven hydrogen production. Int. J. Hydrog. Energy.

[21] Khojasteh F., Mersagh M.R., Hashemipour H. (2022). The influences of Ni, Ag-doped TiO_2_ and SnO_2_, Ag-doped SnO_2_/TiO_2_ nanocomposites on recombination reduction in dye synthesized solar cells. J. Alloy. Compd..

[22] Ou H.H., Lo S.L., Liao C.H. (2011). N-Doped TiO_2_ prepared from microwave-assisted titanate nanotubes (Na_x_H_2-x_Ti_3_O_7_): The effect of microwave irradiation during TNT synthesis on the visible light photoactivity of N-Doped TiO_2_. J. Phys. Chem. C.

[23] Minakshi M., Mitchell D.R.G., Munnangi A.R., Barlow A.J., Fichtner J. (2018). New insights into the electrochemistry of magnesium molybdate hierarchical architectures for high performance sodium devices. Nanoscale.

[24] Wang X.L., Liu L., An H.T., Zhong Y., Wang D.H., Tang C.Y., Hu C.H. (2019). (Sr_0.6_Bi_0.305_)_2_Bi_2_O_7_ as a new visible-light-responsive photocatalyst: An experimental and theoretical study. Mater. Res. Bull..

[25] Zhang W.N., Zhang Q.G., Wang X.H., Yan X.X., Xu J.Q., Zeng Z.G. (2017). Lead-free organic-inorganic hybrid perovskite heterojunction composites for photocatalytic applications. Catal. Sci. Technol..

[26] Feizpoor S., Habibi-Yangjeh A., Vadivel S. (2017). Novel TiO_2_/Ag_2_CrO_4_ nanocomposites: Efficient visible-light-driven photocatalysts with n-n heterojunctions. J. Photochem. Photobiol. A Chem..

[27] Mizoguchi H., Bhuvanesh N.S.P., Woodward P.M. (2003). Optical and electrical properties of the wide gap, n-type semiconductors: ZnBi_2_O_6_ and MgBi_2_O_6_. Chem. Commun..

[28] Wang X.L., Hu C.H., An H.T., Zhu D., Zhong Y., Wang D.H., Tang C.Y., Sun L.X., Zhou H.Y. (2021). Photocatalytic removal of MB and hydrogen evolution in water by (Sr_0.6_Bi_0.305_)_2_Bi_2_O_7_/TiO_2_ heterostructures under visible-light irradiation. Appl. Surf. Sci..

[29] Spadavecchia F., Ardizzone S., Cappelleetti G., Falciola L., Ceotto M., Lotti D. (2013). Investigation and optimization of photocurrent transient measurements on nano-TiO_2_. J. Appl. Electrchem..

[30] Pragathiswaran C., Smitha C., Abbubakkar B.M., Govindhan P.N., Krishnan A. (2021). Synthesis and characterization of TiO_2_/ZnO–Ag nanocomposite for photocatalytic degradation of dyes and anti-microbial activity. Mater. Today Proc..

[31] Ding Y.B., Zhang G.L., Wang X.R., Zhu L.H., Tang H.Q. (2017). Chemical and photocatalytic oxidative degradation of carbamazepine by using metastable Bi^3+^ self-doped NaBiO_3_ nanosheets as a bifunctional material. Appl. Catal. B Environ..

[32] Devi L.G., Kavitha R. (2016). A review on plasmonic metal-TiO_2_ composite for generation, trapping, storing and dynamic vectorial transfer of photogenerated electrons across the Schottky junction in a photocatalytic system. Appl. Surf. Sci..

[33] Wang X., Li Z., Zhang Y., Li Q., Du H., Liu F., Zhang X., Mu H., Duan J. (2022). Enhanced photocatalytic antibacterial and degradation performance by p-n-p type CoFe_2_O_4_/CoFe_2_S_4_/MgBi_2_O_6_ photocatalyst under visible light irradiation. Chem. Eng. J..

[34] Ren F.M., Ma H.H., Hu W., Zhou Z.F., Xu W.B. (2015). Preparation of Sn-doped CdS/TiO_2_/ conducting polymer fiber composites for efficient photocatalytic hydrogen production under visible light irradiation. J. Appl. Polym. Sci..

[35] Xu X., Yang G.R., Liang J., Ding S.J., Tang C.L., Yang H.H., Yan W., Yang G.D., Yu D.M. (2014). Fabrication of one-dimensional heterostructured TiO_2_@SnO_2_ with enhanced photocatalytic activity. J. Mater. Chem. A.

[36] Chen W., Liu T.Y., Huang T., Liu X.H., Duan G.R., Yang X.J., Chen S.M. (2015). A novel yet simple strategy to fabricate visible light responsive C, N-TiO_2_/g-C_3_N_4_ heterostructures with significantly enhanced photocatalytic hydrogen generation. RSC Adv..

[37] Chen H., Li Z.-H., Zhao L., Yang G.-D. (2019). Synthesis of TiO_2_@ZnIn_2_S_4_ hollow nanospheres with enhanced photocatalytic hydrogen evolution. Rare Met..

[38] Low J., Yu J., Jaroniec M., Wageh S., Al-Ghamdi A.A. (2017). Heterojunction photocatalysts. Adv. Mater..

